# Prevalence of pulp and periapical diseases in the endodontic postgraduate program at the national autonomous University of Mexico 2014-2019

**DOI:** 10.4317/jced.60451

**Published:** 2023-06-01

**Authors:** Andrea-Soledad-Sepúlveda Pérez, Enrique-Chávez Bolado, Liliana A. Camacho-Aparicio, Luis-Pablo-Cruz Hervert

**Affiliations:** 1 Endodontic Postgraduate, División de Estudios de Posgrado e Investigación, Facultad de Odontología, Universidad Nacional Autónoma de México, Ciudad Universitaria

## Abstract

**Background:**

Investigations on the prevalence of pulp and periradicular diseases in Mexican population produced few studies, conducted to specific age population. Considering the importance of epidemiological investigation. The aim of this study was to estimate the prevalence of pulp and periapical pathologies and their distribution according to sex, age, affected teeth, and etiological factors found in patients the DEPeI, FO, UNAM Endodontic Postgraduate Program during the period 2014–2019.

**Material and Methods:**

The data collected were from the records of the Single Clinical File of patients treated at the Endodontic Specialization Clinic, DEPeI, FO, UNAM, period 2014–2019. The following variables were recorded for each endodontic file: diagnosed pulp and periapical pathology, sex, age, affected tooth, and etiological factor. Descriptive statistical analysis was performed with 95% CI (Confidence intervals).

**Results:**

Of all the registers reviewed, irreversible pulpitis (34.58%) and chronic apical periodontitis (34.89%) proved to be the most prevalent pulp and periapical pathologies, respectively. The female sex predominated (65.36%). The age group that requested the most endodontic treatment, according to the records reviewed, was 60 or older (36.99%). The most treated teeth were the upper first molars (24.15%) and lower (36.71%), and the most prevalent etiological factor was dental caries (84.07%).

**Conclusions:**

Irreversible pulpitis and chronic apical periodontitis were the most prevalent pathologies. The predominant sex was female, and the age group was 60 years or older. The first upper and lower molars were the most endodontically treated teeth. The most prevalent etiological factor was dental caries.

** Key words:**Pulp pathology, periapical pathology, prevalence.

## Introduction

The pulp tissue reacts against different irritants, predominantly bacteria, through an inflammatory process. Depending on the intensity and duration of the irritant and the resistance of the host, the pathology of the pulp tissue can range from reversible inflammation to severe irreversible inflammation that will lead to necrosis (1-7).

The inflammatory reaction that occurs in the periapical tissues to physical, chemical, and/or bacterial pulp irritants (1-7) can be acute to chronic, depending on the relationship between the host and aggressor agent, which is due to the presence of bacterial toxins and bacteria that reach the periapical tissues through the apical foramen (1-8). This can also occur in other areas of the periodontium due to a lateral canal or in the area of root division through communication between the floor of the pulp chamber and the periodontal tissue (7).

Pulp and/or periapical pathologies vary in prevalence according to the studies carried out in the different countries and in the different populations studied (9-23); the etiology of these diseases varies and can be caused by one or more etiological agents. Biologics (bacteria) are the most common cause of pulp and periapical disease and are generally associated with caries (1,2,3,8,10,12,20,24). In México, investigations carried out by the Oral Pathologies Epidemiological Surveillance System (SIVEPAB) determined that caries is progressively increasing in prevalence, affecting 89.5% of the population over 20 years of age. Of the patients evaluated in the health system, 25% had diseases of the pulp and periapical tissues (25). This situation requires that decision-makers invest in prevention and programs to promote oral health to implement programs that, ideally, depend on the real needs of a population.

Research on the prevalence of pulp and periapical pathologies in the Mexican population is scarce (13,14,23), and they focus on a specific age group or only on pulp pathology. Additionally, in the Endodontics Clinic of the Division of Postgraduate Studies (DEPeI) of the Faculty of Dentistry (FO) of the National Autonomous University of Mexico (UNAM), there was no such study. Considering the importance of research on prevalence, the objective of this study was to estimate the prevalence of pulp and periapical pathologies and their distribution according to sex, age, affected teeth, and etiological factors found in the Postgraduate Endodontics DEPeI, FO, UNAM, period 2014–2019.

## Material and Methods

This was an observational, retrospective study. The Universe for the study had all the clinical records filed in the digital system Single Clinical File (ECU), which has the patient’s anamnesis and contains the endodontic clinical record of the patients treated at the Endodontics Clinic of the Studies Division of Postgraduate and Research (DEPeI), of the Faculty of Dentistry (FO), of the National Autonomous University of Mexico (UNAM), between August 2014 and June 2019. The sampling was nonprobabilistic, for convenience, using the total number of clinical records. Inclusion criteria: Data contained in the electronic ECU system of patients who underwent endodontic treatment at the Postgraduate Endodontics Clinic, FO, UNAM, between August 2014 and June 2019. Exclusion criteria: Electronic single clinical records that were found to be incomplete or lacked any of the study variables (sex, age, affected tooth, etiological factor of the patient, pulp pathology, and periapical pathology).

The variables used in this study were the following: sex, age (age group), affected tooth and etiological factor of the patient (caries, trauma, abrasion, others), pulp and periapical pathologies according to the classification used in endodontics postgraduate (healthy pulp, reversible pulpitis, irreversible pulpitis, pulp necrosis, pulp hyperplasia and pulped tooth), which is based on that performed by Torabinejad and Walton, 2010 and modified by the collegiate body of professors of the Endodontics Specialty of DEPeI, FO, UNAM. The age variable was categorized by age groups that could be analyzed.

The diagnoses issued in the ECU were made according to the signs and symptoms of the patient with extra and intraoral examination, application of pulp sensitivity tests to cold and heat, electrical tests, and, in some cases, special pulp tests such as anesthesia selective, cavity tests, and transillumination. In turn, periapical tests consisting of horizontal and vertical percussion, palpation, mobility, and periodontal probing were carried out. In addition, the dentoalveolar radiograph taken from the affected tooth at the time of its evaluation was analyzed and interpreted. Each diagnosis was validated by a specialist teacher belonging to the Endodontics Postgraduate Program of DEPeI, FO, UNAM.

Before starting any treatment, each resident read to each patient so that they could understand the informed consent. After any doubts were resolved and the patient understood the pertinent explanations to their satisfaction, their signature was requested to continue with the endodontic procedure.

The data obtained from the ECU, which are in an Excel format, were exported to the statistical program STATA V.13.1 and analyzed through descriptive statistics. Then, the distribution and frequency for each variable of this study were determined. Confidence intervals were calculated for each variable at 95% (95% CI). To estimate the prevalence, the total number of records analyzed from the Endodontics Specialty Clinic, DEPeI, FO, UNAM was used as the denominator after applying the exclusion criteria, which added up to a total of 10,990 records.

The study was carried out with the authorization of the Research and Ethics Committee of the School of Dentistry of the National Autonomous University of Mexico.

The data were handled with discretion and in accordance with the rules of respect for personal data. Sensitive data was not used.

In the endodontic clinical file, informed consent includes the acceptance that the data contained in your Clinical Record can be used for research carried out at DEPeI, FO, UNAM. (CIE/0411/11/2019).

## Results

After applying the inclusion and exclusion criteria, 10,990 records of endodontic records from the ECU were analyzed, corresponding to the period August 2014 to June 2019. After performing the statistical analyses, with a 95% confidence interval (95% CI), it was observed that only 6.21% (n = 683) of the records analyzed corresponded to the diagnosis of healthy pulp. The most prevalent pulp pathology was irreversible pulpitis (34.58%, n = 3,800), followed by pulp necrosis (28.67%, n = 3,151). Healthy apical tissues were diagnosed in 38.35% (n = 4,215). The most prevalent periapical pathology was chronic apical periodontitis (34.89%, n = 3,834), followed by acute apical periodontitis (19.97%, n = 2,195). There were no cases of condensing osteitis as shown in Table 1.

**Table 1:**
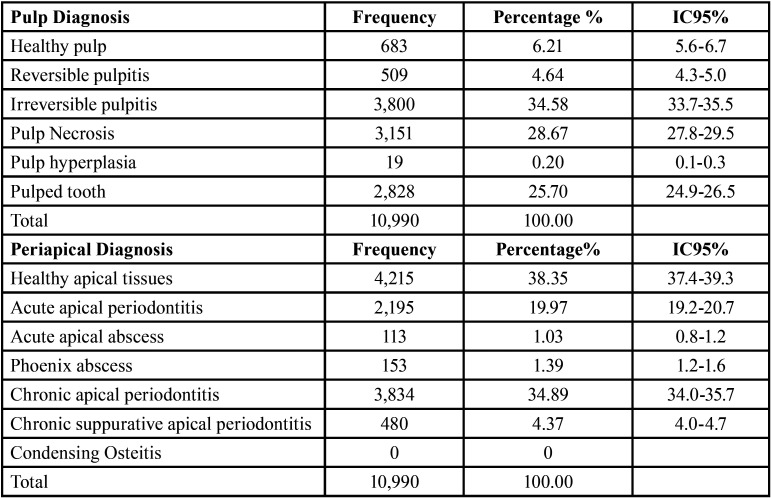
Distribution of pulp and periapical pathologies.

The female sex predominated with 65.36% (n = 7,183). Male 34.64% (n=3,807). In women, irreversible pulpitis occurred in a greater number of cases (n = 2,642, 36.8%), and in men, it was pulp necrosis (n = 1,304, 34.3%), closely followed by irreversible pulpitis (n = 1,158,30.4%). Chronic apical periodontitis was the most prevalent periapical pathology (n = 2,410, 33.6%; n = 1,424.37.4%) in both men and women.

The age group that most requested endodontic treatment, according to the records reviewed, was 60 years or more, with 37.0% (n = 4,065). Followed by groups: 50 to 59 years (23.17%), 40 to 49 years (16.34%), 30 to 39 years (10.64), 19 to 29 years (9.57%), 0 to 18 years (3.28%), (Table 2).

**Table 2:**
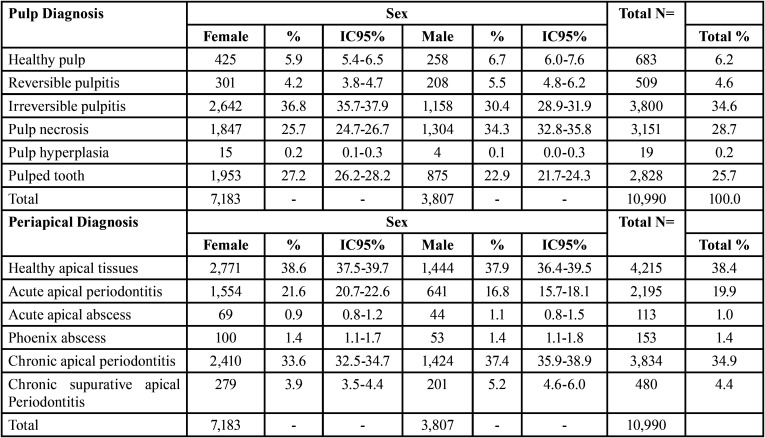
Sex distribution according to pulp and periapical diagnosis.

The age group distribution according to pulp pathology (Table 3) revealed irreversible pulpitis and pulp necrosis as the pathologies that occurred with the highest proportion in the group aged 60 or over (n = 1,237 and n = 1,157, respectively). The diagnosis of healthy apical tissues prevailed in the group aged 60 or over (n = 1867), and the periapical pathology that most affected this group was chronic apical periodontitis (n = 1,322) (Table 3).

**Table 3:**
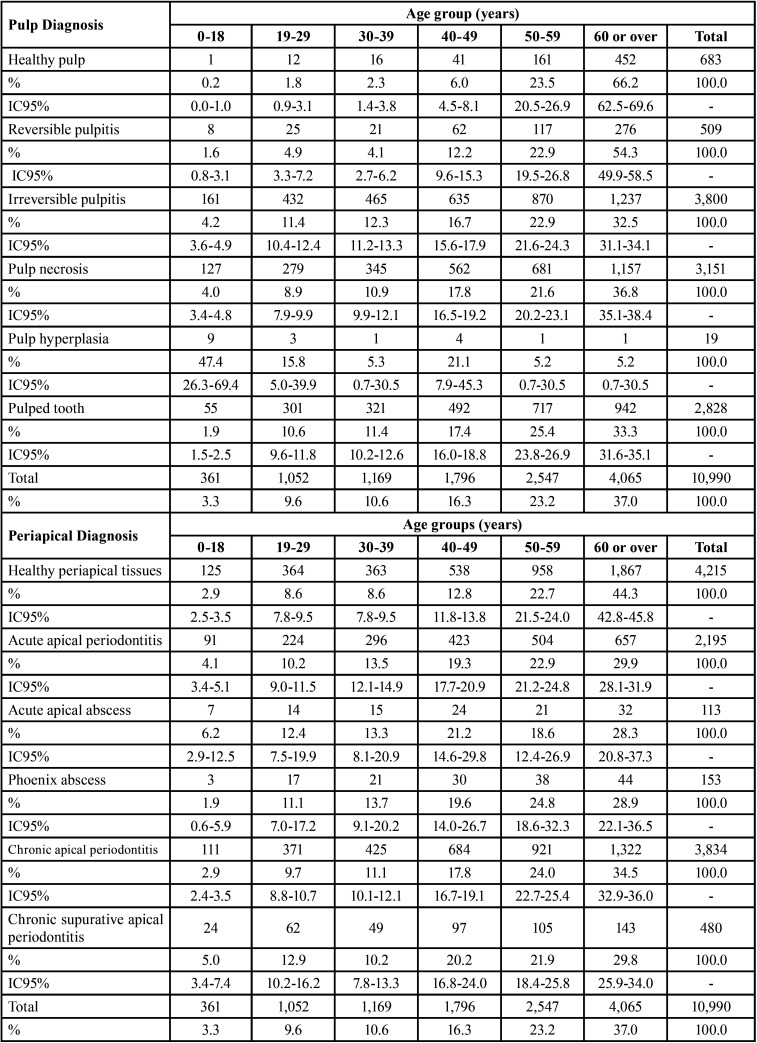
Age distribution according to pulp and periapical diagnosis.

The maxillary teeth were more affected (57.15%), versus mandibular teeth (42.85%). According to the reviewed clinical records, the upper tooth that received the most treatments was the first molar with 24.15% (n = 1,517). For the mandibular dental organs, the first molar was also the most affected, with 36.71% (n = 1,737). The most treated maxillary teeth, which follow the first molar in decreasing order are: 2nd premolar (14.84), central (14.19%), 1st premolar (13.45%), 2nd molar (12.02), lateral (11.81%), canine (9.06 %), 3rd molar (0.41%). The most affected mandibular teeth after the first molar, in decreasing order are: 2nd molar (22.8%), 2nd premolar (13.8%), 1st premolar (9.9%), canine (5.7%), central (5.2%), lateral (4.5%), 3rd molar (1.4%).

Of all the records reviewed, the etiological factor that occurred in most cases was dental caries, with 84.07% (n = 9,239), as shown in Table 4.

**Table 4:**
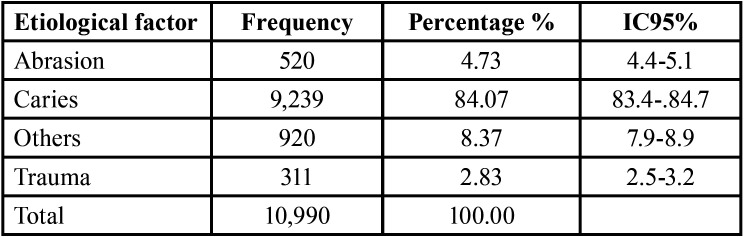
Distribution by etiological factor.

## Discussion

The main objective of this retrospective observational study was to describe the prevalence of pulp and periapical diseases and their distribution according to sex, age, affected teeth, and etiological factors through the analysis of clinical records of patients treated at the Endodontics Clinic of the Division of Postgraduate Studies and Research from the Faculty of Dentistry of the National Autonomous University of Mexico, located in Mexico City, the capital of the country.

Given that the records studied, corresponding to endodontically treated patients in postgraduate studies, are not a random sample, caution must be exercised when trying to extrapolate the data to the Mexican population. However, this endodontic clinic is a reference service in Mexico City and for other states of the country, so it could be similar to the reality of the region.

The results of this investigation show that irreversible pulpitis was the most prevalent pulp pathology. Coinciding with studies such as those carried out in Brazil (9,17), Argentina (10), Mexico (14,23), and Colombia (15). However, our results differ from those found in other investigations, such as those carried out in Cuba, where pulp hyperemia (11) and reversible pulpitis (12) predominated over other pulp pathologies, in Mexico, where reversible pulpitis was the most prevalent disease (13), and in a study carried out in the United States, pulpitis was the most frequent pulp diagnosis (16). These discrepancies may be due to the different methodologies used in the different studies, in addition to the population groups analyzed and the different classifications that were used for both pulp and periapical diagnoses. Age groups also varied in some investigations, in addition to location geographic, added to this the difference in population sizes, which could justify the inequalities found.

The diagnosis of healthy periapice was evidenced in a higher percentage with respect to periapical pathologies, which may be because most of the treated cases corresponded to irreversible pulpitis, which, although it may generate periapical pathology, had not yet been evidenced radiographically, which probably occurred in this investigation. We found that the most prevalent periapical pathology in postgraduate endodontics was chronic apical periodontitis. This disease was also the most prevalent in other investigations, such as those carried out in Brazil (9) and Mexico (13); in this case, the diagnosis of asymptomatic apical periodontitis may be analogous to chronic apical periodontitis, which is why we consider a result similar to that reported in our investigation; likewise, it is reported in the study of Colombia (15) and Venezuela (18). We differ from the studies carried out in Cuba (11,12), where both investigations report that acute apical abscess represents the most prevalent pathology, as does the study carried out in the United States (16). This could be attributed to the fact that they were emergency services where the studies were carried out, that is, they studied the concurrence of patients for emergency pulp and/or periapical treatments, unlike those analyzed in our study, since it studies the entire population that attends for endodontic treatment, either for prosthetic or periodontal indications, among others, in addition to emergency cases. Differences were also evidenced in a study carried out in Mexico (14), in which acute apical periodontitis, such as that found in Brazil (17), was the most prevalent periapical disease. It should be noted that our research differs from several articles found in the literature because they studied the presence or absence of apical periodontitis but did not specify whether apical periodontitis is chronic, chronic suppurative, or acute, so the variation in the results are justified, as found in studies carried out in Argentina (10), Kosovo (20), Scotland (21), and Morocco (22). A study carried out in Brazil (19) noted the difference between apical periodontitis present in teeth without previous endodontic treatment and apical periodontitis in teeth that have previously received endodontic treatment, making evident the difference in percentages, since our study takes all of the nonendodontic cases and divides them based on whether they have received previous endodontic treatment.

The predominant sex in this research was female, with 65.36% (n = 7,183). This could be attributed to the fact that women go more frequently for dental care and are more concerned about their oral health. In addition, this may be because a large part of this population group has more time to go at the times established by the endodontics specialty because they do not have a paid job with hours that prevent them from going to their appointments, contrary to a large number of men, who found it difficult to attend appointments. What we found in this research was similar to that reported by various investigations (9,10,11,13,16-19,23) but differs from other studies where the male sex was preponderant, as found in Cuba (12) or in Scotland (21). Irreversible pulpitis occurred more frequently in women and pulp necrosis in men, followed by irreversible pulpitis. Studies such as those carried out by Oliveira *et al*. (9) also analyzed the distribution of pathologies according to sex, agreeing that irreversible pulpitis occurred more frequently in women but highlighting that it is symptomatic and that in men, it is asymptomatic irreversible pulpitis that most occurred, differing in terms of what was obtained in this study. This also shows the difference in terms of the classification used in this research, which does not refer to a difference between symptomatic and asymptomatic patients. Another study that considers sex according to the distribution of pathologies is that of Quiñones (11), which does not agree with what was found in the present study, since pulp hyperemia was the predominant pulp disease in women and pulpitis in men. Acute purulent may be another classification considered for pulp and periapical lesions based on the sample size and the methodology used. The differences found with the reviewed articles may be due to the methodologies of the studies and to the different populations studied.

The age group that was most affected in this research was that of patients aged 60 or over; this group was also the most affected with irreversible pulpitis and chronic apical periodontitis, corresponding to the most prevalent diseases analyzed in this study. This age group could have a greater predominance over the others due to the deterioration of oral health with age; therefore, they may require more dental treatments, including endodontic treatments, either due to cavities or some prosthetic indication or periodontal problems. Likewise, this population group has more time available for dental care, so this factor could also be attributed to making it the age group that was most attended to in postgraduate endodontics. Studies such as those carried out in Yucatán, Mexico (14) and Kosovo (20) report that the age group most affected is those aged 60 or over, as was observed in this study. However, in this variable analyzed, we found more discrepancies with what was observed in other studies, most of them being younger age groups. This may be due to the different age groups studied in the different investigations and to the different methodologies and population groups. Different age groups have been reported in these studies: 22 to 29 years (11), 33 to 45 years (12), over 45 years (15), 29 to 48 years (17), under 45 years (18), 40 to 49 years (19), and 46 to 55 years (21). As seen, the groups are very varied, and this can also be attributed to the fact that the way of analyzing the variable was diverse in the different reports; that is, the age group is not comparable, since they are not distributed in the same way as analyzed in this research.

The maxillary teeth were affected in a greater proportion than the mandibular teeth. From this analysis, it was found that for both upper and lower teeth, the first molars were the most treated. For this study, the first molars were the most affected; in turn, several studies report similar results (13,14,15,17,22,23). However, we found discrepancies with investigations that describe the upper incisors as the dental organs that required the greatest number of endodontic treatments (9,18,19). It should be noted that after the incisors, the molars were seen as the most affected teeth in these studies. This may be because they are the first permanent posterior teeth to erupt and, therefore, are affected by cavities on many occasions due to lack of proper hygiene and high consumption of cariogenic foods, among others. It is also the most reported tooth with endodontic treatment failure, which increases the number of cases for these molars, specifically the upper first molar, due to its high anatomical complexity.

The etiological factor for most of the cases analyzed in this research was dental caries, with 84.07%, corresponding to that reported in the worldwide literature, identifying caries as the most prevalent factor causing pulp and periapical pathologies. This is evidenced by studies such as those carried out in Argentina (10), Cuba (12), Colombia (15), Venezuela (18), and Morocco (22). It should be noted that there are several investigations that do not analyze etiological factors; therefore, we cannot compare them with our study.

Dental caries is the predominant bacterial irritant in the Mexican population, according to studies carried out by the System of Epidemiological Surveillance of Oral Pathologies (SIBEVAP), which causes pulp and periapical effects (25).

## Conclusions

In this research, the most prevalent pulp pathology was irreversible pulpitis, and the periapical pathology was chronic apical periodontitis. The predominant sex in this study was female, with irreversible pulpitis occurring more frequently in women and pulp necrosis in men.

The age group of 60 years or older was the most affected, with irreversible pulpitis and chronic apical periodontitis mainly present in this group.

The maxillary teeth presented a greater number of cases than the mandibular teeth. The upper and lower first molars were the most endodontically treated teeth.

The etiological factor that caused most of the pulp and periapical diseases was dental caries.
